# Supplementary material for: Accurate and Efficient Phonon Calculations in Molecular Crystals via Minimal Molecular Displacements
Source: J Chem Theory Comput. 2025 Jun 17;21(16):8073–85. doi: 10.1021/acs.jctc.5c00494 (PMC12392453; doi:10.1021/acs.jctc.5c00494)
Supplement: Supplementary file 1 [file ct5c00494_si_001.pdf]

Supporting Information for: Accurate and Efficient Phonon Calculations in Molecular Crystals via Minimal Molecular Displacements

Lorenzo Soprani,[†,¶] Andrea Giunchi,[‡,¶] Marco Bardini,[†] Quintin N. Meier,[†] and Gabriele D'Avino*[,†,§]

†*Grenoble Alpes University, CNRS, Grenoble INP, Institut Néel, 38042 Grenoble, France*

‡*CINECA National Supercomputing Center, Casalecchio di Reno, I-40033 Bologna, Italy*

¶*Dipartimento di Chimica Industriale "Toso Montanari", Università di Bologna, 40129 Bologna, Italy*

§*Department of Molecular Sciences and Nanosystems, Ca' Foscari University of Venice, Venice, Italy*

E-mail: gabriele.davino@unive.it

Note S1: Transformation between atomic and molecular coordinates

In Eq. 2 of the main text we introduced the set of molecular-displacement coordinates as a linear combinations of Cartesian atomic displacements

$$u_j = \sum_i U_{ij}\, x_i. \tag{S1}$$

A similar relationship holds between mass-weighted (tilde superscript) molecular and atomic displacements

$$\tilde{u}_j = \sum_i \widetilde{U}_{ij}\, \tilde{x}_i. \tag{S2}$$

We provide here an operative description of how the U and $\widetilde{U}$ matrices are built in our method. U can be written as the concatenation of three matrices

$$U = (U_{\mathrm{T}}, U_{\mathrm{R}}, U_{\mathrm{V}}) \tag{S3}$$

where the sub-matrices U_{T}, U_{R}, U_{V} refer to molecular translations (T), rotations (R) and vibrations (V), respectively.

The U_{T} block contains the translation vectors of each molecule along its principal inertia

axes

$$U_{\mathrm{T}} = \begin{pmatrix} \frac{\boldsymbol{X}^{(1)}}{\sqrt{N_{\mathrm{a}}^{(1)}}} & & & \\ \vdots & & & \\ \frac{\boldsymbol{X}^{(1)}}{\sqrt{N_{\mathrm{a}}^{(1)}}} & & & \\ & \frac{\boldsymbol{X}^{(2)}}{\sqrt{N_{\mathrm{a}}^{(2)}}} & & \\ & \vdots & & \\ & \frac{\boldsymbol{X}^{(2)}}{\sqrt{N_{\mathrm{a}}^{(2)}}} & & \\ & & \ddots & \\ & & & \frac{\boldsymbol{X}^{(N_{\mathrm{m}})}}{\sqrt{N_{\mathrm{a}}^{(N_{\mathrm{m}})}}} \\ & & & \vdots \\ & & & \frac{\boldsymbol{X}^{(N_{\mathrm{m}})}}{\sqrt{N_{\mathrm{a}}^{(N_{\mathrm{m}})}}} \end{pmatrix}, \tag{S4}$$

where $\boldsymbol{X}^{(i)}$ and $N_{\mathrm{a}}^{(i)}$ represent the 3×3 matrix of the inertia tensor eigenvectors and the number of atoms of the ith molecule, respectively. Vertical dots indicate that the 3×3 blocks are replicated for the $N_{\mathrm{a}}^{(i)}$ atoms. N_{m} is the number of molecules in the unit cell or supercell.

The U_R block describes the rotation of each molecule about its principal inertia axes

$$U_\mathrm{R} = \boldsymbol{N} \begin{pmatrix} \boldsymbol{P}_1^{(1)} & & & & \\ \vdots & & & & \\ \boldsymbol{P}_{N_\mathrm{a}^{(1)}}^{(1)} & & & & \\ & \boldsymbol{P}_1^{(2)} & & & \\ & \vdots & & & \\ & \boldsymbol{P}_{N_\mathrm{a}^{(2)}}^{(2)} & & & \\ & & \ddots & & \\ & & & \boldsymbol{P}_1^{(N_\mathrm{m})} & \\ & & & \vdots & \\ & & & \boldsymbol{P}_{N_\mathrm{a}^{(N_\mathrm{m})}}^{(N_\mathrm{m})} & \end{pmatrix}, \tag{S5}$$

where $\boldsymbol{N}$ is a diagonal matrix that normalizes the columns of U_R and $\boldsymbol{P}_i^{(j)}$ are 3×3 matrices. The 3 columns of $\boldsymbol{P}_i^{(j)}$ corresponds the rotation tangent vectors for the ith atom of the jth molecule, each column corresponding to one of the inertia axes about which rotations are performed.

Finally, the U_V block is built from single-molecule vibrational analysis. Specifically, we start from single-molecule Cartesian normal modes $\{\mathbf{q}^{(i)}\}$:

$$U_\mathrm{V} = \begin{pmatrix} \mathbf{q}_1^{(1)} & \cdots & \mathbf{q}_{N_\mathrm{v}^{(1)}}^{(1)} & & & & & & \\ & & & \mathbf{q}_1^{(2)} & \cdots & \mathbf{q}_{N_\mathrm{v}^{(2)}}^{(2)} & & & \\ & & & & & & \ddots & & \\ & & & & & & & \mathbf{q}_1^{(N_\mathrm{m})} \cdots & \mathbf{q}_{N_\mathrm{v}^{(N_\mathrm{m})}}^{(N_\mathrm{m})} \end{pmatrix}, \tag{S6}$$

The V subspace requires, however, additional attention. Indeed, upon transforming U as obtained from equations Eq. S3-S6 to mass-weighted coordinates (c.f. Eq. 8 in the

main text), one obtains a non-orthogonal basis of mass-weighted displacements, i.e. the $\widetilde{U}$ matrix is not unitary, although deviations from unitarity for the investigated systems were found to be very small. This arises from the different molecular geometries between the molecules in the crystal (used to build rotations about inertia axes) and the optimized geometry employed in the isolated-molecule normal mode analysis. Given the convenience of working with an orthogonal basis of mass-weighted coordinates, we perform a Gram-Schmidt orthonormalization, which projects out rotations from the V subspace, leaving T and R blocks unchanged. This leads to a unitary $\widetilde{U}$ matrix. The final U employed to build displaced structures in solid-state calculations is thus obtained from the corrected (unitary) $\widetilde{U}$ matrix by inverting Eq. 8 in the main text.

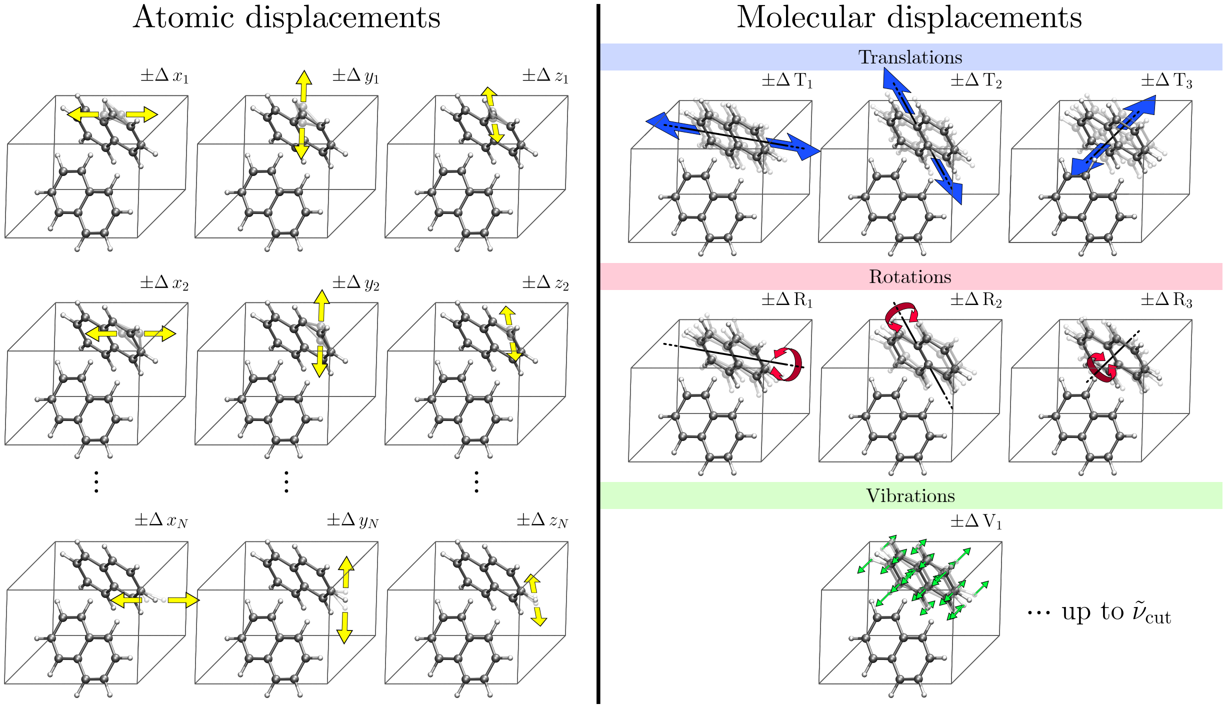

Figure S1: Graphic representation of atomic and molecular displacements.

Table S1: Cell parameters.

System	a (Å)	b (Å)	c (Å)	α (°)	β (°)	γ (°)
Naphthalene	7.777	5.933	8.080	90.000	114.071	90.000
Pentacene	6.239	7.636	14.330	76.978	88.136	84.415
BTBT	11.849	5.881	8.031	90.000	106.261	90.000
C4-BTBT-C4	4.643	7.652	13.444	86.187	82.177	84.247

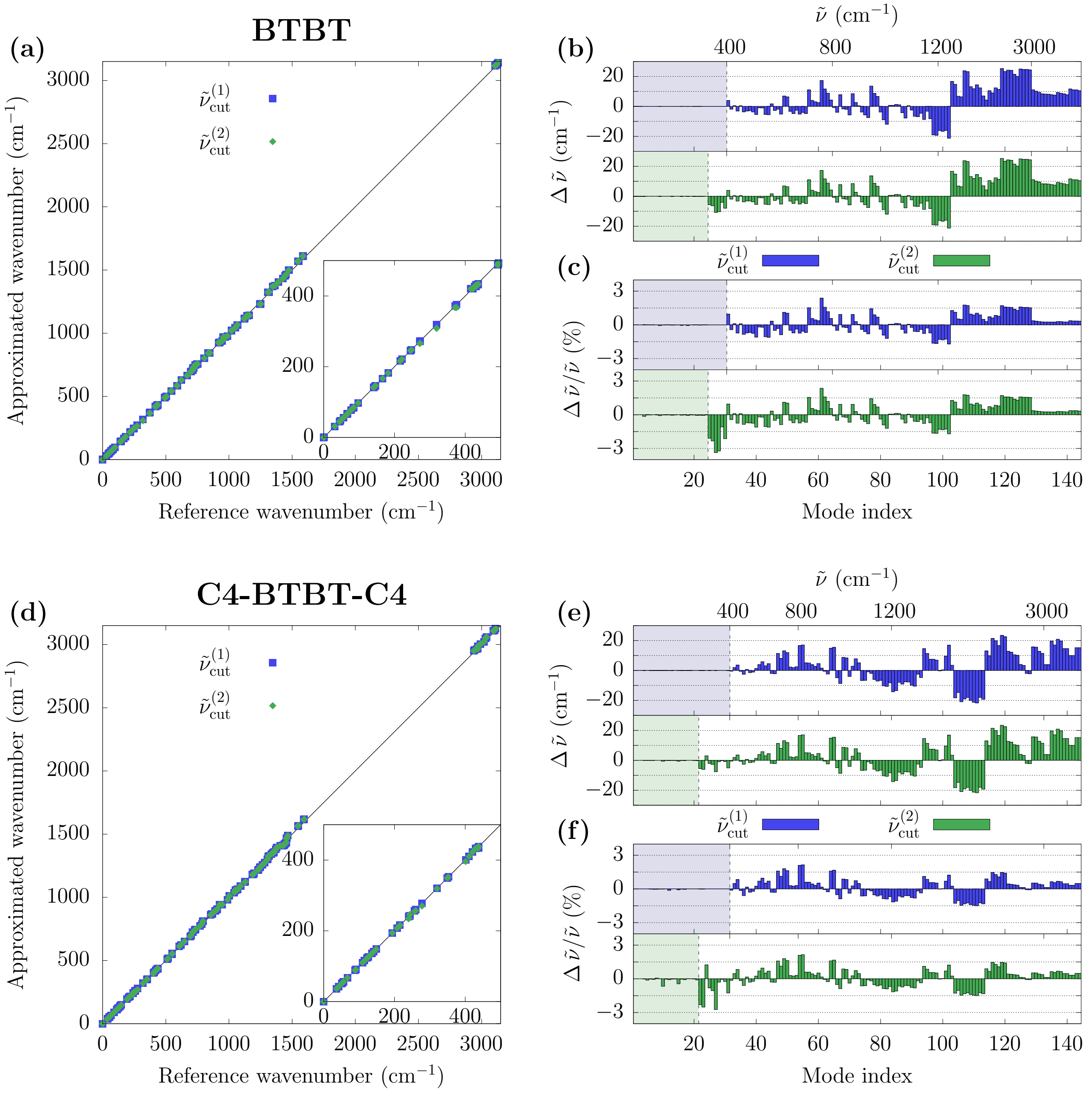

Figure S2: Comparison between the exact and MMD-approximated phonon frequencies for the BTBT (panel a-c) and C4-BTBT-C4 (panels d-f) crystals. MMD results refer to approximated dynamical matrices built with $\tilde{\nu}^{(1)}_{\mathrm{cut}}$ and $\tilde{\nu}^{(2)}_{\mathrm{cut}}$ cutoff criterion for the selection of V_L modes. (a,d) Plots of the exact vs. approximated frequencies. The insets provide a zoom of the low-frequency region. (b,e) Difference and (c,f) relative difference between MMD and reference vibrational frequencies. Values are reported against the mode index, ranked in ascending frequency order. An approximate top x-scale reporting mode frequencies is provided as an indication.

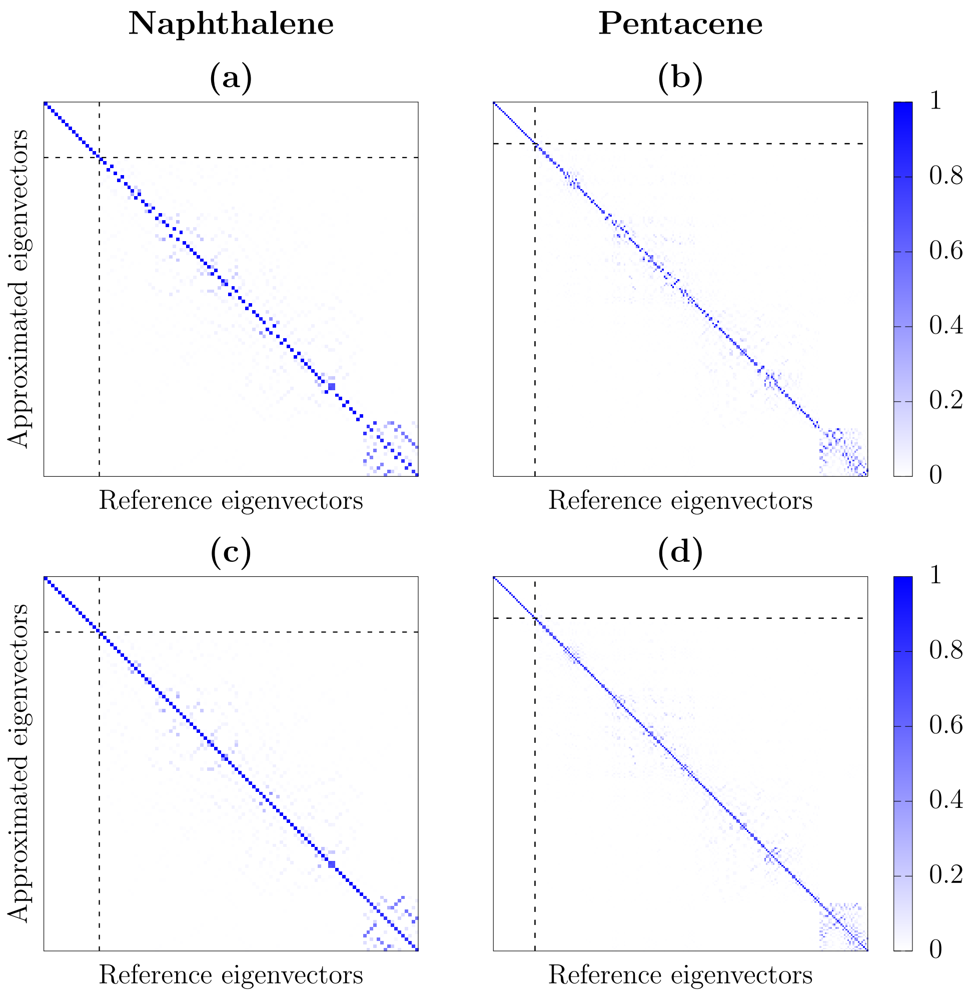

Figure S3: Overlap matrices between the Γ point reference and MMD-approximated eigenvectors for (a,c) naphthalene and (b,d) pentacene. MMD results have been obtained with the cutoff frequency $\tilde{\nu}_{\mathrm{cut}}^{(1)}$. The eigenvectors in the top panels (a,b) are sorted in ascending frequency order. Bottom panels (c,d) match reference and approximated eigenvectors according to a maximum overlap criterion. The dashed lines delimitated the low and high frequency regions. The dimension of the upper-left quadrant corresponds to the number of modes for which displacements are calculated with solid-state DFT.

Table S2: List of **k** point meshes used to sample the Brillouin zone in the Monkhorst-Pack scheme.

System	Unit cell			Supercell $2 \times 2 \times 2$		
Naphthalene	3	3	2	2	2	1
Pentacene	2	2	1	1	1	1
BTBT	2	3	2	1	2	1
C4-BTBT-C4	3	2	1	2	1	1

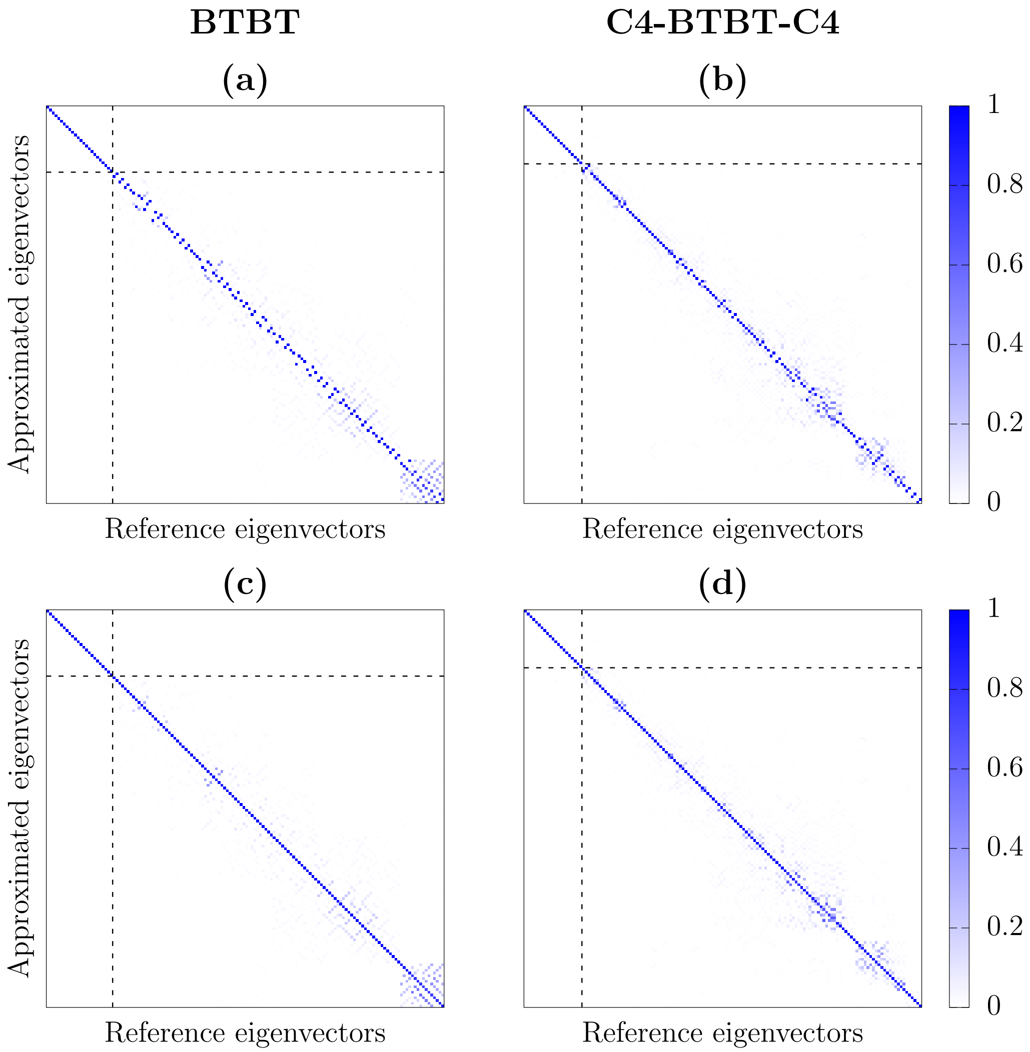

Figure S4: Same of Figure S3 for for (a,c) BTBT and (b,d) C4-BTBT-C4.

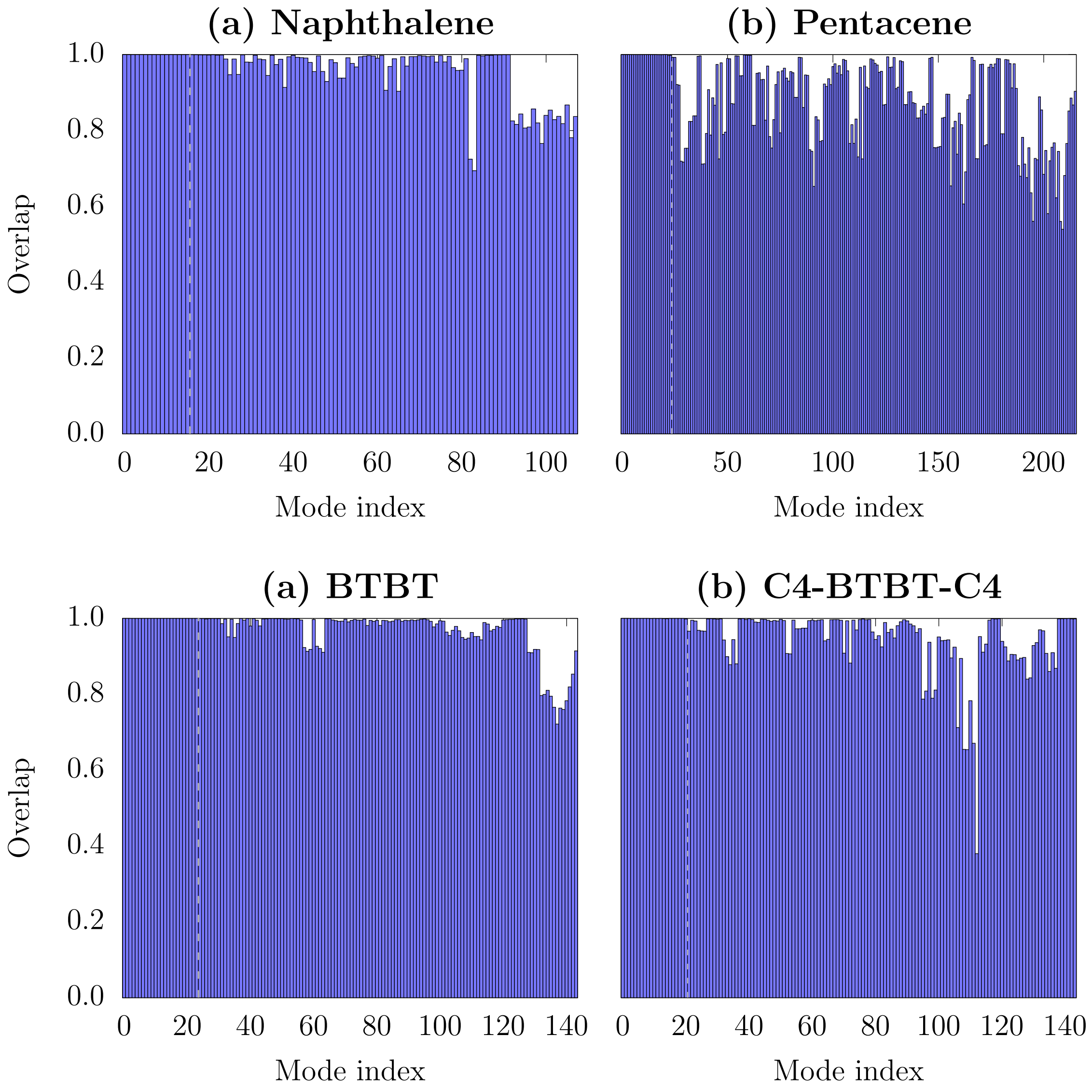

Figure S5: Overlap between reference and MMD (cutoff $\tilde{\nu}_{\mathrm{cut}}^{(1)}$) phonon eigenvectors. The dashed line marks the boundary between the first $Z(6+N_{\mathrm{a}})$ modes (i.e. the numer of the displaced geometries for which forces are actually computed with expensive solid-state DFT calculations) and the others.

Table S3: High symmetry points in the first Brillouin zone defining the paths adopted in phonon bands plots.

System	Space group	Point	a^*	b^*	c^*
Naphthalene BTBT	$P2_1/c$ (14)	Z	0.0	0.5	0.0
		Γ	0.0	0.0	0.0
		A	−0.5	0.0	0.5
		E	−0.5	0.5	0.5
		Y	0.5	0.0	0.0
Pentacene C4-BTBT-C4	P-1 (2)	X	0.5	0.0	0.0
		Γ	0.0	0.0	0.0
		Y	0.0	0.5	0.0
		R	0.5	0.5	0.5
		Z	0.0	0.0	0.5

Table S4: List of organic semiconductor molecules with their number of atoms (N_a) and the number of isolated molecule vibrational modes below $200\,cm^{-1}$.

System	N_a	N_V
naphthalene	18	2
anthracene	24	2
BTBT	24	4
tetracene	30	5
DNTT	36	7
pentacene	36	6
hexacene	42	8
MO-pyrene	42	12
MT-pyrene	42	12
C4-BTBT-C4	48	14
fuDT	48	12
MT-perylene	48	16
F-NDI-Cl_2	50	23
MT-peropyrene	56	17
rubrene	70	19
C8-BTBT-C8	72	26
dF-TESADT	76	30
C8-DNTT-C8	84	29

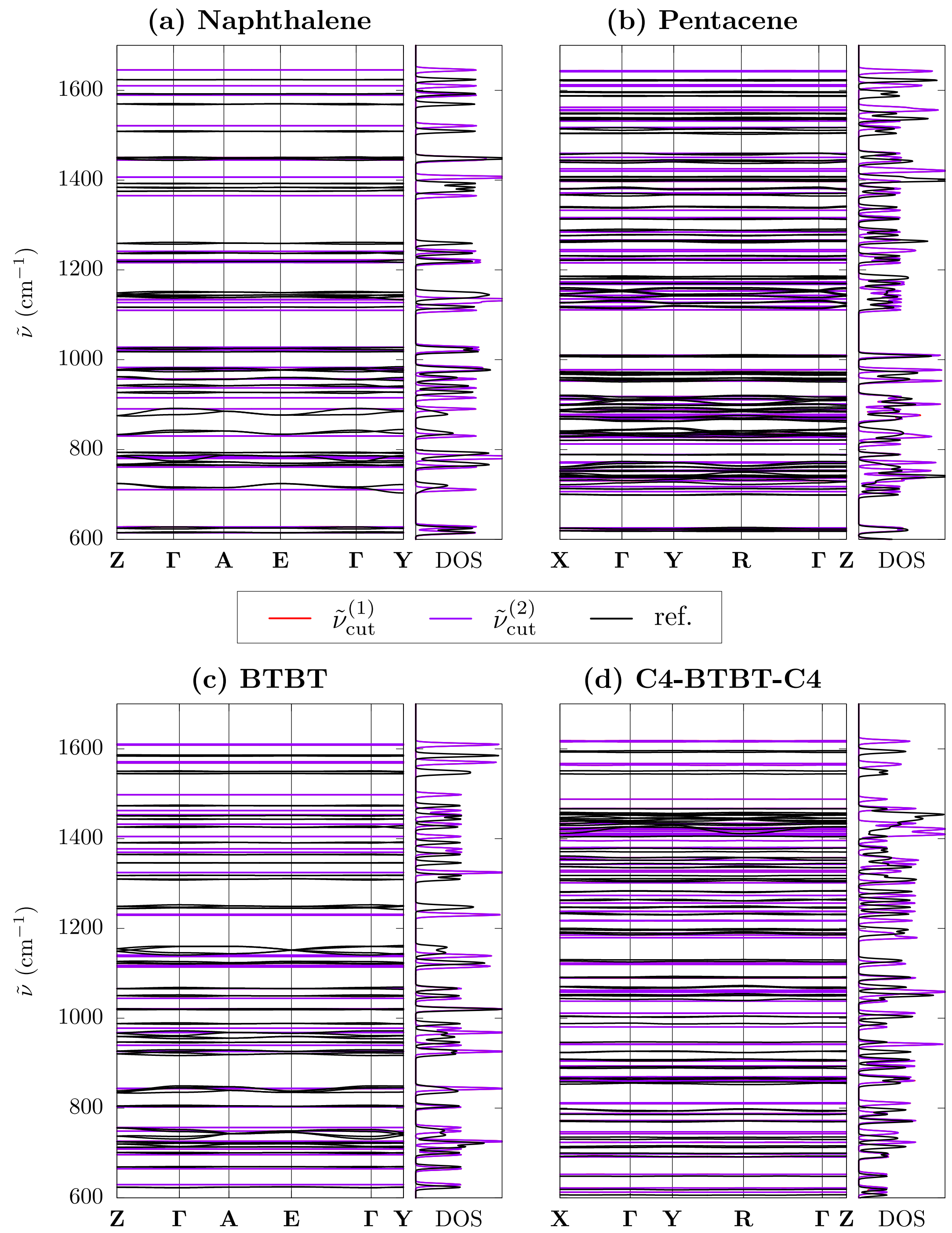

Figure S6: Phonon band structures and DOSs at frequencies above 600 cm^{-1}. The region of C-H vibrations is omitted for clarity. The black lines are reference data, the purple and the red ones (the latter hidden by the former) are the results obtained with the MMD method. Phonon bands and DOSs obtained for $\tilde{\nu}_{\rm cut}^{(1)}$ and $\tilde{\nu}_{\rm cut}^{(2)}$ are the same in this frequency range.
